# Intramedullary Stress and Strain Correlate with Neurological Dysfunction in Degenerative Cervical Myelopathy

**DOI:** 10.3390/app15020886

**Published:** 2025-01-17

**Authors:** Mahmudur Rahman, Karthik Banurekha Devaraj, Omkar Chauhan, Balaji Harinathan, Narayan Yoganandan, Aditya Vedantam

**Affiliations:** Department of Neurosurgery, Medical College of Wisconsin, Milwaukee, WI 53226, USA

**Keywords:** degenerative cervical myelopathy (DCM), cervical spondylotic myelopathy (CSM), finite element modeling, intramedullary stress and strain, neurological dysfunction, decompression surgery

## Abstract

Degenerative cervical myelopathy (DCM) is characterized by progressive neurological dysfunction, yet the contribution of intramedullary stress and strain during neck motion remains unclear. This study used patient-specific finite element models (FEMs) of the cervical spine and spinal cord to examine the relationship between spinal cord biomechanics and neurological dysfunction. Twenty DCM patients (mean age 62.7 ± 11.6 years; thirteen females) underwent pre-surgical MRI-based modeling to quantify von Mises stress and maximum principal strains at the level of maximum spinal cord compression during simulated neck flexion and extension. Pre-surgical functional assessments included hand sensation, dexterity, and balance. During flexion, the mean intramedullary stress and strain at the level of maximum compression were 7.6 ± 3.7 kPa and 4.3 ± 2.0%, respectively. Increased intramedullary strain during flexion correlated with decreased right-hand sensation (r = −0.58, *p* = 0.014), impaired right-hand dexterity (r = −0.50, *p* = 0.048), and prolonged dexterity time (r = 0.52, *p* = 0.039). Similar correlations were observed with intramedullary stress. Patients with severe DCM exhibited significantly greater stress during flexion than those with mild/moderate disease (*p* = 0.03). These findings underscore the impact of dynamic spinal cord biomechanics on neurological dysfunction and support their potential utility in improving DCM diagnosis and management.

## Featured Application

Patient-specific finite element models of degenerative cervical myelopathy (DCM) patients correlate with neurological function, assess disease severity, and can potentially guide personalized surgical planning.

## 1. Introduction

Degenerative cervical myelopathy (DCM) is the most common cause of spinal cord dysfunction in older adults and an important cause of disability and impaired quality of life [1]. Intramedullary stress and strain during neck motion are known contributors to axonal and myelin injury in DCM [2]. However, it is not known if intramedullary stress and strain are linked to neurological dysfunction in DCM. This critical knowledge gap has limited our understanding of the clinical relevance and potential utility of incorporating spinal cord biomechanics into clinical decision-making for DCM patients. Although the direct measurement of intramedullary stress and strain is not feasible in humans, methods to estimate intramedullary stress and strain are expected to improve the evaluation of ongoing spinal cord damage, thereby boosting the accuracy of the diagnosis and management of DCM.

Validated finite element models (FEMs) of the human cervical spine provide a non-invasive assessment of clinical spine biomechanics in relation to its normal biomechanical function (intrinsic-cord-related and external-range-of-motion-related load transfer), injury, degeneration, or surgery [3–5]. A FEM is a computational method used to predict the behavior of materials under external mechanical loading, simulating physiological activities [6,7]. Advances in computational capabilities and engineering software have broadened the applications of FEMs in medicine. In spine surgery, FEMs have been used to quantify implant biomechanics, evaluate load distributions in spinal structures, and predict the biomechanical effects of surgical interventions on spinal alignment and stability under physiological conditions [8–10]. Additionally, advancements in 3D modeling have enabled the development of patient-specific anatomical models from imaging modalities like MRI and CT.

Prior approaches to the 3D FEM of the cervical spinal cord, however, have used a generic spinal cord model with geometric and material properties from animal and cadaver spinal cord tissue [11–14]. These generic models do not incorporate the specific geometries of the individual patient’s cervical spine, which are well-known contributors to intramedullary stress and strain [15,16]. While a comprehensive review of patient-specific and generic FEMs is beyond the scope of this study, prior review articles have highlighted challenges faced by previous studies in developing patient-specific models. Some of these challenges include a limited scope for studying generic responses, a lack of advanced imaging tools to obtain accurate patient-specific geometries, and in some cases, limited interdisciplinary collaboration between clinical personnel and modeling teams, which are often based in engineering disciplines [17–21].

The lack of patient-specific data in these models has limited the clinical translation of biophysical modeling. Patient-specific FEMs can quantify intramedullary stress and strain for individual patients more accurately and have greater potential for clinical translation. However, no prior studies have directly linked patient-specific FEM outputs to neurological dysfunction in DCM patients. This study addresses this critical gap by utilizing patient-specific FEMs to determine the relationship between pre-surgical spinal cord biomechanics and pre-surgical neurological function in DCM. By incorporating individualized spinal cord geometries, we aim to assess how intramedullary stress and strain correlate with functional impairments and clinical severity in DCM.

## 2. Materials and Methods

We prospectively recruited 20 DCM patients between the ages of 18 and 90 who were scheduled for cervical spine surgery at a single academic medical center. All patients were clinically diagnosed with DCM by a board-certified spine surgeon. For enrollment in the study, the diagnosis of DCM included the following features: (1) one or more signs of myelopathy (gait ataxia, positive Romberg’s sign, inability to tandem walk, corticospinal motor deficits, hand atrophy, hyperreflexia, a positive Hoffman sign, upgoing plantar reflexes, or lower limb spasticity); (2) one or more symptoms of myelopathy (impaired hand dexterity, numb hands, gait impairment, bilateral hand paresthesia, L’Hermitte’s phenomena, and/or weakness in arms or legs); and (3) evidence of cervical spinal cord compression during magnetic resonance imaging (MRI). Any subjects unable to tolerate MRI for any reason were also excluded. We excluded patients with a history of trauma, syringomyelia, prior cervical spine surgery, spinal cord hemorrhage, tumor, pregnancy, and patients with other neuromuscular diseases that could explain their symptoms. Institutional review board approval was obtained, and all participants provided written informed consent.

### 2.1. Generation of Patient-Specific Surface Model from MRI

Patient-specific FEMs of the osteo-ligamentous spine were created from their pre-surgical MRI. A 3T General Electric Premier MRI Scanner was used to acquire a T2-weighted fast spin echo image with a 3D isotropic resolution of 0.8 × 0.8 × 0.8 mm with a T_R_/T_E_ of 2500/122 ms. Sagittal MRI was used to record the spinal cord level of maximum compression and grade intervertebral disc degeneration at all cervical spine levels [22]. Vertebral body and spinal cord surface geometries were developed from the DICOM images using the Mimics v26.0 (Materialise NV, Leuven, Belgium) software package. Surface patches were applied to delineate the endplate and facet regions. The MRI-derived surface geometry was exported as computer-aided design geometry.

### 2.2. Finite Element Modeling of Cervical Spine

**Figure 1.**
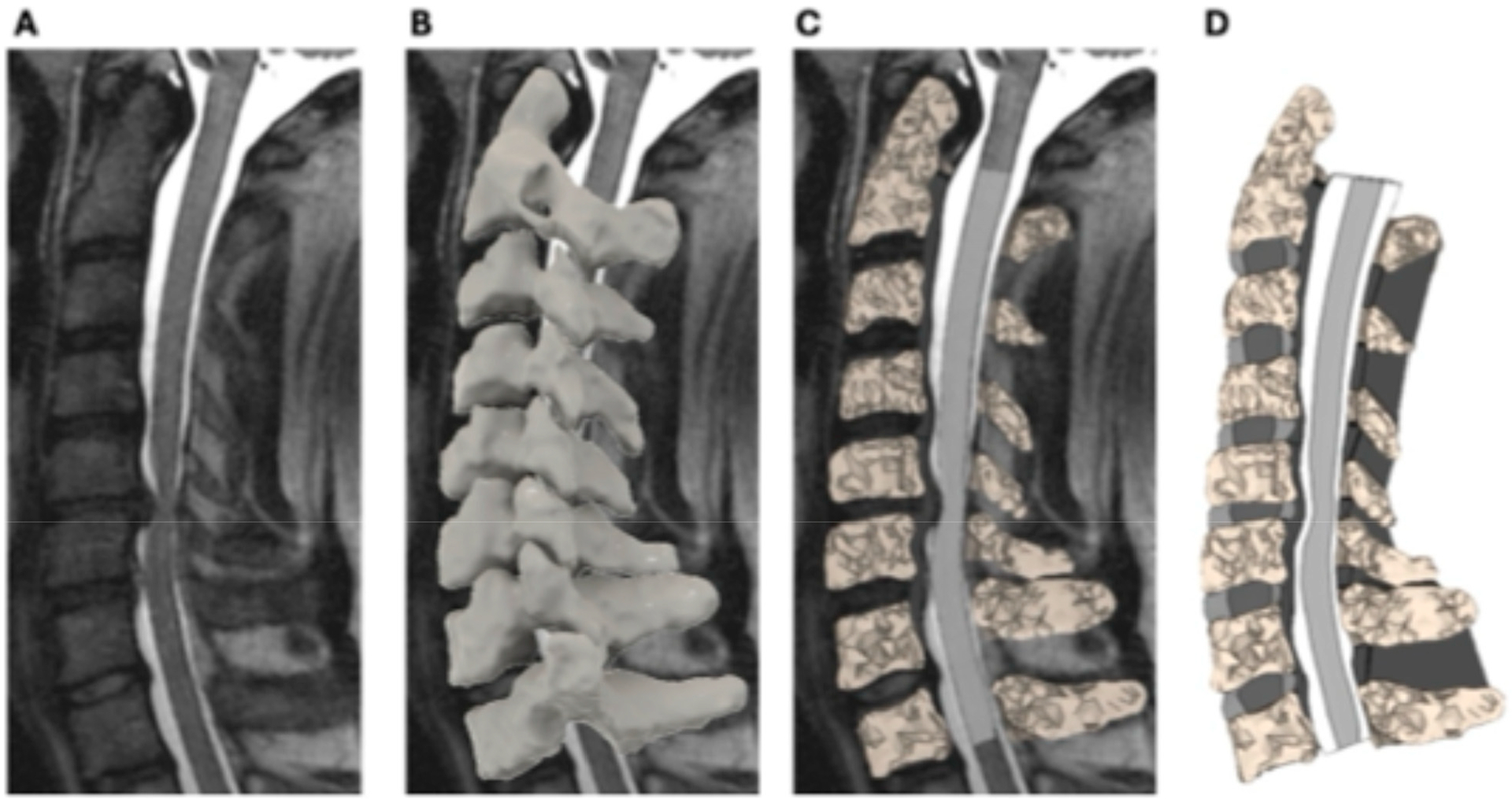
Illustration of the patient-specific finite element modeling of the cervical spine and spinal cord. Sagittal T2-weighted MRI of the cervical spine showing spinal cord compression at the C5–C6 level (**A**). MRI data were imported into Mimics software (Materialise NV, Leuven, Belgium) to generate a 3D surface model of the cervical spine (**B**). Mesh representation of the finite element model, illustrating segmentation of the vertebrae, intervertebral discs, and spinal cord for biomechanical analysis (**C**,**D**).

The 3D cervical spine surface model was imported into ANSA software v22.1.0 (BETA CAE Systems, Farmington Hills, MI, USA) for finite element meshing. T2-weighted MRI was used to grade disk degeneration at each level [22], and the corresponding disk properties were incorporated into the model. The spinal cord FEM model consisted of spinal cord parenchyma, pia mater, dura mater, denticulate ligaments, and cerebrospinal fluid [15]. The model used material properties from human spinal cord tissue that have been reported previously [15]. This patient-specific spinal cord FEM was integrated with the cervical spine model for analysis (Figure 1).

### 2.3. Loading and Boundary Conditions

**Figure 2.**
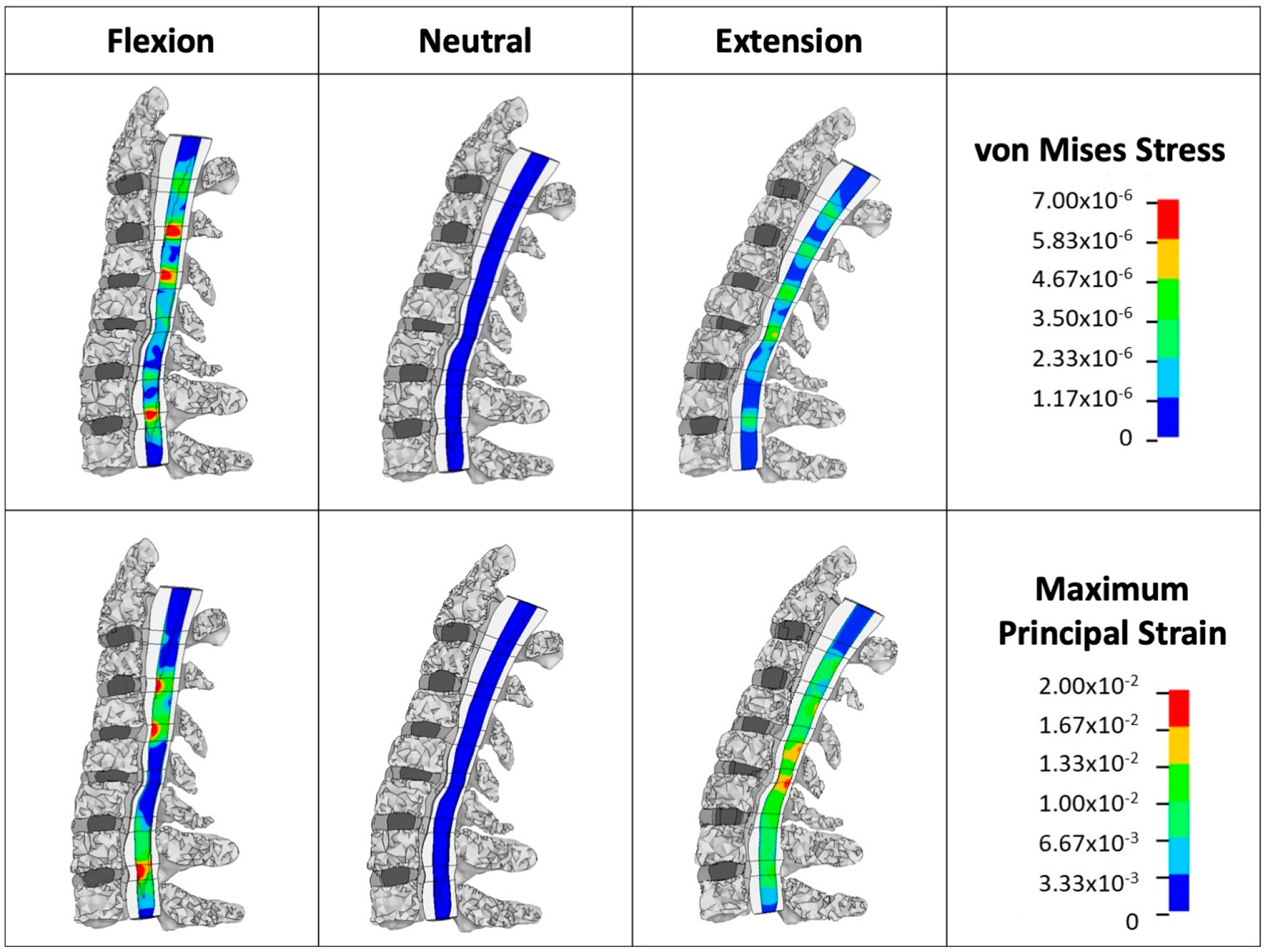
Finite element model simulation: loading and boundary condition illustration of applied boundary conditions and loading in the patient-specific FEM during simulated neck flexion and extension. Sagittal loadings of 2 Nm and a follower force of 75 N simulate head mass and muscle forces. Segmental von Mises stress (VMS) and maximum principal strain (MPS) were calculated for the spinal cord under these conditions.

The patient-specific FEMs were constrained at the inferior surface of the T1 vertebra in all degrees of freedom, and the sagittal loadings were applied at the superior vertebra. The pure moment load level was 2 Nm each in the flexion and extension modes. An additional follower force of 75 N to simulate the head’s mass and muscle force was applied during flexion and extension using the method described by Patwardhan et al. [23]. The 75 N loading was maintained using LS-PrePost v4.3 and LS-Dyna v9.0.1 (Livermore, CA, USA). Motion-related biomechanical outputs, including the segmental range of motion (ROM), segmental von Mises stress (VMS), and maximum principal strain (MPS) in the spinal cord, were calculated (Figure 2). MPS and vMS at the level of maximum spinal cord compression, as well as the maximum MPS and vMS across the entire cervical spinal cord, were recorded.

### 2.4. Assessment of Neurological Function

All DCM participants underwent pre-surgical functional testing. Functional measures included the modified Japanese Orthopedic Association scale (mJOA) and Myelopathy Disability Index (MDI), both myelopathy-specific symptom surveys, and the Quick Disabilities of Arm, Shoulder, and Hand (QuickDASH), which surveys upper limb dysfunction during activities of daily living. The Berg Balance Scale (BBS) was used to assess balance. Short Form 36 Physical Component Score version 2 (SF36 PCS) was used to assess quality of life. Upper limb strength, sensation, and dexterity were measured using the Graded Redefined Assessment of Strength, Sensibility, and Prehension Version-Myelopathy (GRASSP-M) [24]. Handgrip strength and pinch strength were assessed separately for the right and left hands using a Jamar Plus Hand Dynamometer and Jamar Digital Pinch Gauge, respectively. The average of three trials for each hand was used for analysis.

### 2.5. Statistical Analysis

Descriptive statistics were used for demographics and pre-surgical functional scores. FEM outputs and neurological function were related using partial correlations controlling for age. The statistical significance of each relationship was calculated using two-tailed hypothesis tests for the correlation coefficients (r), with significance set at *p* < 0.05. Statistical analysis was performed using MATLAB (Boston, MA, USA), SPSSv23 (IBM, Armonk, NY, USA), and statistical significance was set at *p* < 0.05.

## 3. Results

### 3.1. Demographics and Pre-Surgical Functional Scores

A total of 20 participants (7 males and 13 females) with a mean age of 62.7 ± 11.6 years were included in this study. Patient demographic information and pre-surgical functional survey scores are summarized in Table 1.

### 3.2. Pre-Surgical Intramedullary Stress and Strain

**Figure 3.**
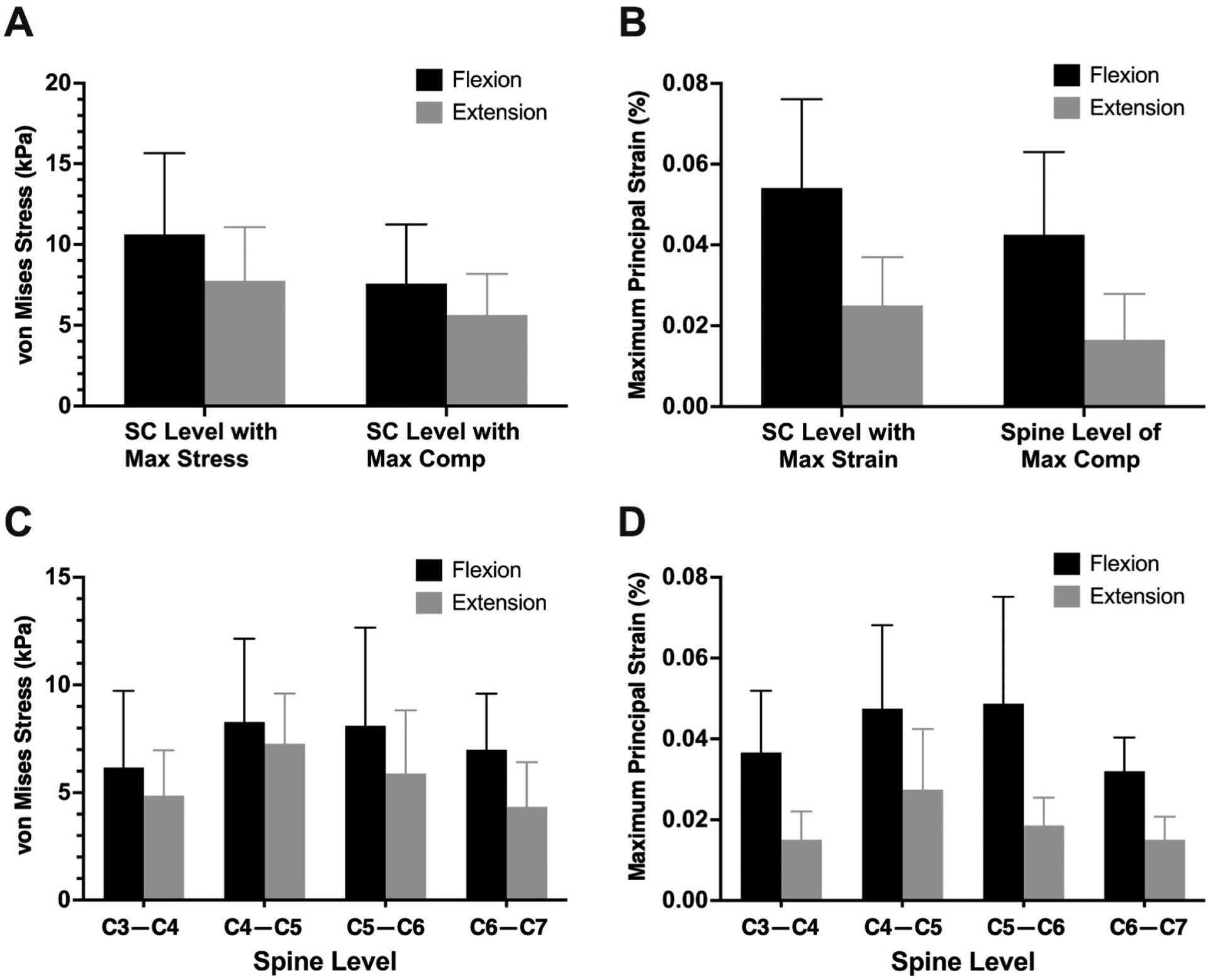
Mean intramedullary von Mises stress (**A**) and principal strain (**B**) during neck flexion and extension from patient-specific finite element models. Mean von Mises stress (**C**) and principal strain (**D**) at each cervical spine level during neck flexion and extension. Error bars represent standard deviations.

The mean intramedullary stress and strain at the level of maximum compression during flexion was 7.57 ± 3.66 kPa and 0.04 ± 0.02, respectively. Across subjects, the maximum stress/strain in the cervical spinal cord during flexion was 10.6 ± 5.04 kPa and 0.05 ± 0.02, respectively. The mean intramedullary stress and strain at the level of maximum compression during extension was 5.63 ± 2.56 kPa and 0.02 ± 0.01, respectively. Across subjects, the maximum stress/strain in the cervical spinal cord during extension was 7.7 ± 3.32 kPa and 0.03 ± 0.011, respectively (Figure 3). The most common cervical spine level of maximum compression was C5–C6 (40%, n = 8), followed by C6–C7 (25%, n = 5), C4–C5 (20%, n = 4), and C3–C4 (15%, n = 3). In 40% of patients (n = 8), the level of maximum compression coincided with the level of maximum von Mises stress during flexion. The stress and strain during flexion and extension are summarized in Table 2.

### 3.3. Association Between Spinal Cord Biomechanics and Pre-Surgical Neurological Function

**Figure 4.**
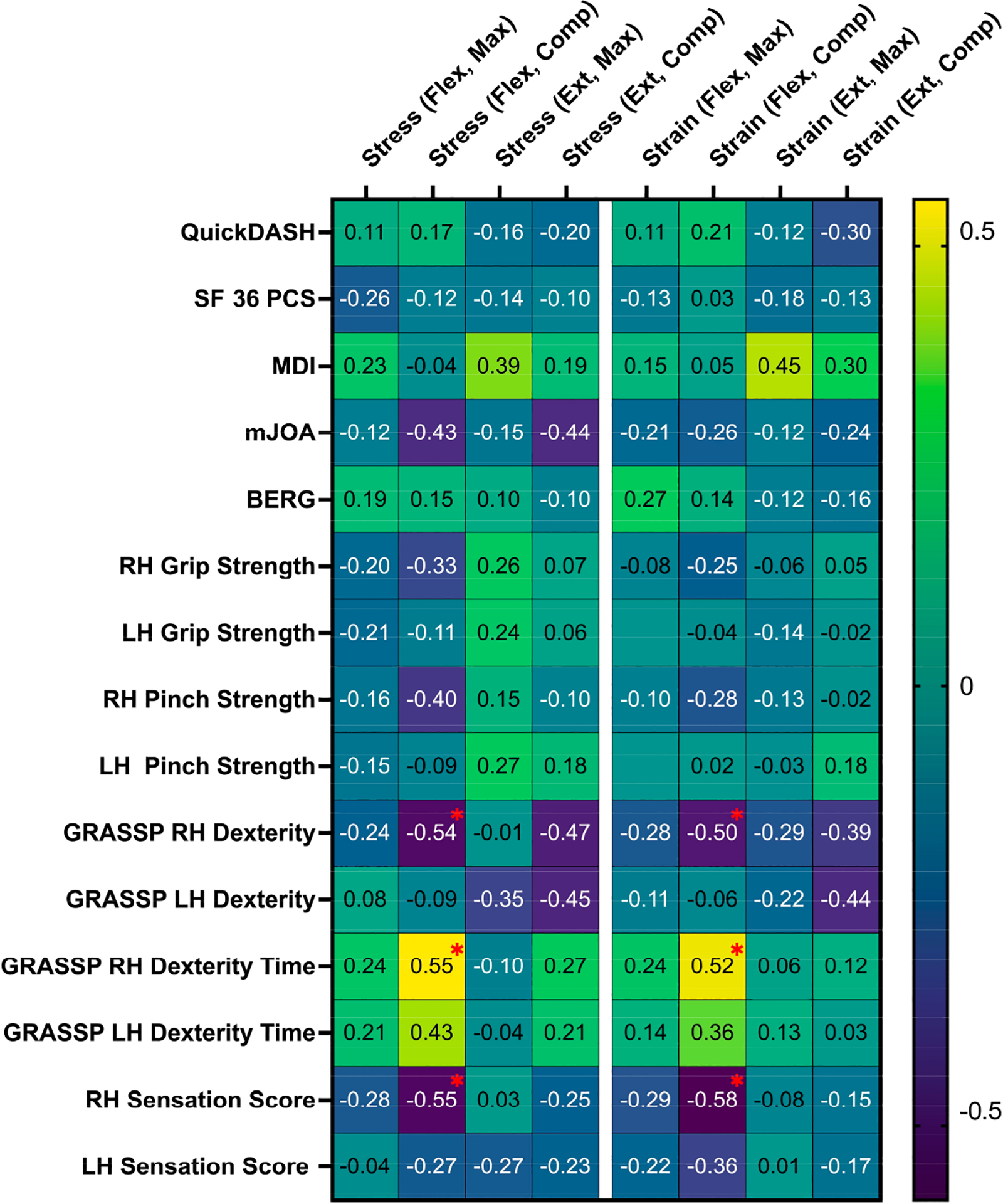
Partial correlation coefficient (r) matrix relating intramedullary stress and strain under flexion and extension with the neurological function of DCM patients. Statistically significant correlations (*p* < 0.05) are highlighted with a red asterisk. Abbreviations: RH, right hand; LH, left hand; QuickDASH, Quick Disabilities of the Arm, Shoulder, and Hand; SF 36 PCS, Short Form 36 Physical Component Score; MDI, Myelopathy Disability Index; mJOA, Modified Japanese Orthopedic Association scale; BERG, Berg Balance Scale; GRASSP, Graded Redefined Assessment of Strength, Sensibility, and Prehension; Flex, flexion; Max, maximum; Comp, compression; Ext, extension.

Increased intramedullary strain during flexion at the level of maximum compression was correlated with decreased right-hand sensation (r = −0.58, *p* = 0.014), decreased right-hand dexterity (r = −0.50, *p* = 0.048), and increased right-hand dexterity time (r = 0.52, *p* = 0.039). Increased intramedullary stress at the level of maximum cord compression during flexion was associated with decreased right-hand sensation (r = −0.55, *p* = 0.023), decreased right-hand dexterity (r = −0.536, *p* = 0.032), and increased right-hand dexterity time (r = 0.55, *p* = 0.026). The correlation coefficients between spinal cord biomechanics and pre-surgical neurological function are summarized in Figure 4. Spinal cord biomechanics was able to discriminate between DCM severity groups. Intramedullary stress at maximum spinal cord compression during flexion was significantly greater for patients with severe DCM (11.1 ± 4.4 kPa) compared to those with mild/moderate DCM (6.7 ± 3.0 kPa, *p* = 0.03).

## 4. Discussion

This study demonstrates for the first time the relationship between intramedullary stress/strain and neurological dysfunction in degenerative cervical myelopathy. Intramedullary stress and strain at the level of maximum spinal cord compression during neck flexion were significantly correlated with sensorimotor hand dysfunction and disease severity. The results of this study highlight the potential clinical utility of spinal cord biomechanics for the diagnoses of degenerative cervical myelopathy.

There is increasing evidence that intrinsic spinal cord forces due to the stress and strain of the cord during neck motion are important contributors to neural injury in DCM [2]. Human cadaver studies have shown that longitudinal stretch (strain) along the spinal cord during flexion is accentuated in the presence of spinal cord compression or kyphosis [25]. Neck motion creates chronic, repetitive stretch injuries that produce neural injury. Evidence for cellular pathways that mediate stretch-associated axonal [26] and myelin [27] injury further supports a larger role for intramedullary stress and strain in the pathophysiology of DCM. The contribution of spinal cord biomechanics to the structural spinal cord suggests a possible mechanism by which intramedullary stress and strain relate to neurological dysfunction in DCM.

Although extrinsic spinal cord compression contributes to spinal cord damage, there is increasing evidence that intrinsic spinal cord forces due to the stress and strain of the cord during neck motion are important contributors to neural injury in DCM [2]. During neck flexion, increased spinal cord strain at the level of maximum cord compression is noted in the dorsal half of the spinal cord affecting the dorsal column–medial lemniscus pathway, which relates to light touch sensation and proprioception relative to the brain. We found that flexion-related intramedullary stress and strain were correlated to hand sensation, and this link is potentially due to the effect of neck flexion on the dorsal columns. In addition, we found that higher pre-surgical intramedullary stress and strain are linked to decreased hand dexterity and prolonged dexterity time. The corticospinal tracts, which control hand dexterity, are located in the dorsal half of the spinal cord, and these tracts experience increased spinal cord tension during neck flexion. Increased tension or deformation in these tracts may disrupt the precision and speed of neural transmission necessary for coordinated hand movements. Together, neck flexion is correlated with sensorimotor dysfunction in DCM due to increased intramedullary stress and strain at the level of maximum cord compression.

There are several possible pathophysiological mechanisms that affect the dorsal spinal cord during neck flexion in DCM patients. Vascular disruption occurs as neck flexion induces elongation and tension in the posterior spinal arteries, leading to a zigzagging pattern and transient reductions in lumen size, which diminish blood flow and increase the risk of ischemic damage to the dorsal columns [28–30]. This is compounded by increased intramedullary pressure due to the flexion-related deformation of the spinal cord, which impedes microcirculatory efficiency and could provoke ischemia within the dorsal neural tissues, disrupting the functionality of the dorsal columns and corticospinal tracts [31–33]. Moreover, the mechanical tension exerted on the dorsal spinal cord can distort mechanosensitive ion channels [34] and the axonal and myelin [35,36] structures within these pathways, impairing axoplasmic transport and leading to sensory and motor dysfunctions. Additionally, shear stresses from pathological flexion or torsional movements significantly impact the dorsal spinal cord, misaligning or displacing neural tissues, which adversely affects the integrity and function of the dorsal columns and associated neural pathways [37,38].

The findings of this study highlight the potential clinical utility of spinal cord biomechanics in DCM. DCM is characterized by clinico-radiological discordance, where DCM patients with substantial neurological dysfunction may have minimal cord compression [39–41]. This can contribute to delays in diagnosis and difficulties with the prognostication of the post-surgical recovery of functions. In these patients, the quantification of spinal cord biomechanics may potentially support an earlier diagnosis and improve the accuracy of predicting post-surgical function. Spinal cord biomechanics can also assist with the diagnosis of certain forms of cervical myelopathy, such as Hirayama’s disease [42], where dynamic spinal cord compression is the primary contributor to neurological dysfunction. The quantification of patient-specific spinal cord biomechanics can also be used for surgical planning. In those patients with high intramedullary stress and strain during neck flexion, motion-preserving surgeries such as cervical laminectomy or laminoplasty are unlikely to provide durable benefits compared to fusion surgeries that will reduce adverse spinal cord tension during neck motion. Our previous work has demonstrated that patient-specific FEMs can be successfully developed for the post-surgical spine, enabling longitudinal assessments of spinal cord biomechanics [15,43,44]. Assessing the link between post-surgical spinal cord biomechanics and function is a goal of future studies.

The limitations of this study include its small sample size, which restricts generalizability and reduces statistical power; a larger sample size would address these issues. Our FEMs also did not differentiate between gray and white matter, as separate gray and white matter properties for the human spinal cord are currently not available. In future studies, we will include patient-specific axial rotation, as well as lateral bending, for a more comprehensive understanding of spinal cord mechanics. Our study is also limited by the lack of patient-specific spinal cord tissue properties in FEMs. Deriving patient-specific tissue properties of the spinal cord is not feasible given the risk of profound neurological damage, and cadaver spinal cord specimens may not reflect true in vivo tissue properties. One technique is the use of advanced microstructural MRI, such as diffusion tensor imaging (DTI). DTI can provide an estimate of spinal cord microstructure, and in our ongoing studies, we are evaluating the link between spinal cord DTI and tissue biomechanics. These studies may create a pathway for non-invasively estimating patient-specific tissue properties to improve the accuracy of patient-specific FE modeling. Despite these limitations, this is the largest study to perform patient-specific cervical spinal cord FEM, and the first to correlate FEM outputs to the patient-reported measures of neurological function.

## 5. Conclusions

Intramedullary stress and strain contribute to neurological dysfunction in degenerative cervical myelopathy (DCM). This study demonstrates an association between increased stress and strain during neck flexion and pre-surgical impairments in sensory and motor function. These findings emphasize the role of dynamic mechanical forces in DCM and support the potential clinical value of biomechanical modeling. By integrating these spinal cord biomechanics into patient assessments, patient-specific FEMs may improve diagnostic precision and inform surgical strategies.

## Figures and Tables

**Table 1.** Demographics and Functional Scores.

	Measure	Mean (SD)	n
Patient Demographics	Age	62.7 (11.6)	20
	Female sex, n (%)	7 (35%)	20
Preoperative Survey Scores	Modified Japanese Orthopedic Association	13.3 (2.08)	20
	Short Form 36 Physical Component Score	35.16 (8.979)	19
	Myelopathy Disability Index	4.6 (2.8)	20
	Quick Disabilities of Arm, Shoulder, and Hand	39.46 (21.9)	19
	Berg Balance Scale	48.15 (7.59)	20
GRASSP * Assessments	Dexterity—right hand	8.1 (1.2)	17
	Dexterity—left hand	8.25 (0.5)	16
	Dexterity time—right hand (s)	43.5 (23.0)	17
	Dexterity time—left hand (s)	47.2 (21.15)	16
	Sensation—right hand	11.4 (1.1)	18
	Sensation—left hand	11.4 (1.0)	18
Grip Strength	Right-hand grip strength (lbs)	80.6 (33.5)	20
	Left-hand grip strength (lbs)	87.1 (39.6)	20
Pinch Grip	Right pinch grip (lbs)	9.45 (4.6)	20
	Left pinch grip (lbs)	10.0 (4.68)	20

Graded Redefined Assessment of Strength, Sensibility, and Prehension Version-Myelopathy.

**Table 2.** Mean Intramedullary stress and strain from patient-specific finite element models (n = 20) during flexion and extension.

FEM Measurement (Units)	Neck Motion	Spinal Cord Level	Mean (SD)
Intramedullary Stress (kPa)	Flexion	Maximum Stress	10.6 (5.04)
		Maximum Compression	7.6 (3.66)
	Extension	Maximum Stress	7.7 (3.32)
		Maximum Compression	5.6 (2.56)
Intramedullary Strain (%)	Flexion	Maximum Strain	0.05 (0.02)
		Maximum Compression	0.04 (0.2)
	Extension	Maximum Strain	0.03 (0.011)
		Maximum Compression	0.02 (0.011)

## Data Availability

The data presented in this study are available upon request from the corresponding author.
